# Prämaligne, zystische Neoplasien und neuroendokrine Tumoren des Pankreaskopfes – Ist die Kausch-Whipple-Resektion eine adäquate Therapie?

**DOI:** 10.1007/s00104-024-02070-5

**Published:** 2024-04-03

**Authors:** Hans G. Beger

**Affiliations:** c/o Universitätsklinikum Ulm, Albert-Einstein-Allee 23, 89081 Ulm, Deutschland

**Keywords:** Zystische Neoplasie, Neuroendokrine Tumore, Pankreatoduodenektomie, Tumorenukleation, Duodenumerhaltende Resektion des Pankreaskopfes, Cystic neoplasm, Neuroendocrine tumor, Pancreatoduodenectomy, Tumor enucleation, Duodenum-preserving resection of the pancreatic head

## Abstract

Das gegenwärtig am häufigsten angewendete operative Verfahren bei symptomatischen, benignen, prämalignen zystischen und neuroendokrinen Tumoren des Pankreaskopfs ist die Whipple-Operation oder pyloruserhaltende Pankreatoduodenektomie (PD); die PD ist jedoch eine Multiorganresektion, bei der pankreatisches und extrapankreatisches Gewebe sowie dessen Funktionen verloren gehen. Die PD ist auch bei benignen Neoplasien mit dem Risiko erheblicher Komplikationen in der frühen postoperativen Phase assoziiert und geht mit einer Krankenhausmortalität von bis zu 5 % einher. Nach Whipple-Operation wird bei 14–20 % der Patienten ein sich neu manifestierender Diabetes mellitus beobachtet, bei 25–45 % tritt eine exokrine Insuffizienz auf, was zu einer nach Resektion benigner Tumoren persistierenden metabolischen Dysfunktion und Beeinträchtigung der Lebensqualität führt. Symptomatische Neoplasien sind Indikation für die operative Therapie. Patienten mit asymptomatischen Pankreastumoren werden gemäß den Kriterien von Surveillance-Protokollen behandelt. Ziel der operativen Therapie bei asymptomatischen Patienten ist entsprechend den Leitlinienkriterien der Abbruch des Surveillance-Programms, bevor sich eine fortgeschrittene Krebserkrankung im Zusammenhang mit der Neoplasie entwickelt. Die Tumorenukleation und duodenumerhaltende Pankreaskopfresektion, entweder total oder partiell, sind parenchymsparende Resektionen bei benignen Neoplasien des Pankreaskopfs. Erste Wahl bei kleinen Tumoren ist die Enukleation; allerdings ist diese mit einem erhöhten Risiko für Pankreasfisteln von Grad B + C nach Pankreas-Hauptgangverletzung assoziiert. Vorteile der duodenumerhaltenden totalen oder partiellen Pankreaskopfresektion sind geringe operationsbedingte Komplikationen, eine Mortalität von < 0,5 % und die Aufrechterhaltung der endo- und exokrinen Pankreasfunktionen. Parenchymsparende Pankreaskopfresektionen sollten die klassischen Whipple-Operationen bei Neoplasien des Pankreaskopfs ablösen.

## Abstract (en)

Currently, the most frequently used surgical treatment for symptomatic, benign, premalignant cystic and neuroendocrine neoplasms of the pancreatic head is the Whipple procedure or pylorus-preserving pancreatoduodenectomy (PD). However, when performed for treatment of benign tumors, PD is a multiorgan resection involving loss of pancreatic and extrapancreatic tissue and functions. PD for benign neoplasm is associated with the risk of considerable early postoperative complications and an in-hospital mortality of up to 5%. Following the Whipple procedure a new onset of diabetes mellitus is observed in 14–20% and new exocrine insufficiency in 25–45%, leading to metabolic dysfunction and impairment of quality of life persisting after resection of benign tumors. Symptomatic neoplasms are indication for surgery. Patients with asymptomatic pancreatic tumors are treated according to the criteria of surveillance protocols. The goal of surgical treatment for asymptomatic patients is, according to the guideline criteria, interruption of the surveillance program before the development of an advanced stage cancer associated with the neoplasm. Tumor enucleation and duodenum-preserving pancreatic head resection, either total or partial, are parenchyma-sparing resections for benign neoplasms of the pancreatic head. The first choice for small tumors is enucleation; however, enucleation is associated with an increased risk of pancreatic fistula B + C following pancreatic main duct injury. Duodenum-preserving total or partial pancreatic head resection has the advantage of low postoperative surgery-related complications, a mortality of < 0.5% and maintenance of the endocrine and exocrine pancreatic functions. Parenchyma-sparing pancreatic head resections should replace classical Whipple procedures for neoplasms of the pancreatic head.

Zystische Tumoren des Pankreas werden häufig diagnostiziert; klinisch relevant davon sind zystische, prämaligne Neoplasien und neuroendokrine Tumoren, die über ein inhärentes Potenzial zur malignen Transformation verfügen. Ziele der operativen Therapie sind Tumorentfernung und Beschwerdefreiheit vor dem Stadium eines Karzinoms und ein sehr niedriges Operationsrisiko. Alternativen zur klassischen Whipple-Resektion gutartiger Neoplasien des Pankreaskopfes sind parenchymsparende lokale Resektionen [1].

## Risiko einer malignen Transformation zystischer Neoplasien

Benigne Tumoren des Pankreas, zystische, prämaligne und neuroendokrine Neoplasien werden infolge Routineanwendung hochauflösender, bildgebender Diagnoseverfahren zunehmend häufiger auch als asymptomatischer Zufallsbefund entdeckt. Intraduktale, papillär-muzinöse Neoplasie (IPMN), muzinös-zystische Neoplasie (MCN) sowie solid-pseudopapilläre Neoplasie (SPN) sind die häufigsten benignen zystischen Tumoren mit inhärentem Risiko einer malignen Transformation [2]. Das Risiko eines IPMN-assoziierten Adenokarzinoms ist in operativen Serien bei Hauptgang- und „Mixed-type“-IPMN ca. 42–65 % [3], bei Seitengang-IPMN 16–46 % [4] und bei MCN 4–17 % [5]. Bei SPN findet sich in der Resektathistologie in 12–18 % ein Malignom [6]. Serös-zystische Adenome (SCN), prädominant im Pankreaskopfbereich lokalisiert, werden selten mit einem Adenokarzinom gefunden. Neuroendokrine Neoplasien (PNETs) machen ca. 2 % aller Pankreastumoren aus, davon sind 65 % nichtfunktionelle, sporadische Tumoren. Funktionelle und nichtfunktionelle (f-/nf‑)PNETs sind heterogene Neoplasien mit variabel malignem Potenzial. Das Risiko einer malignen Transformation ist in operativen Serien von f‑PNETs ca. 30–80 % [7] und bis zu 90 % bei nf-PNETs [8].

## Indikation und Ziele der operativen Therapie

Eine Indikation zur operativen Therapie besteht bei allen Patienten mit einer Neoplasie, die klinische Symptome verursacht. Asymptomatische Hauptgang- und Mixed-type-IPMN des Pankreaskopfes sollten operativ entfernt werden, wenn der Patient für die chirurgische Belastung fit ist [9, 10]. Patienten mit symptomfreien, zufällig diagnostizierten zystischen und neuroendokrinen Neoplasien werden primär in Surveillance-Protokollen überwacht, um eine unnötige Operation zu vermeiden, auch weil die klassischen Operationsverfahren mit einem deutlichen Komplikationsrisiko und metabolischer Langzeitmorbidität verbunden sind. Ein Wechsel zur chirurgischen Therapie bei asymptomatischen Patienten mit Abbruch der Überwachung erfolgt, wenn CT/MRT/MRCP und EUS oder die Punktionshistologie Zeichen einer malignen Transformation erkennen lassen. Kriterien für einen Wechsel zur operativen Therapie sind Tumorgröße und -wachstum, Auftreten muraler Knoten in der Zyste, Pankreasgangerweiterung > 5 mm oder Nachweis einer hochgradigen Dysplasie in der Punktionshistologie bei IPMN oder MCN [9, 10]. Bei funktionellen symptomatischen PNETs besteht eine Indikation zur operativen Therapie. Asymptomatische nf-PNETs werden primär in Surveillance-Protokollen überwacht; eine chirurgische Therapie erfolgt abhängig von Tumorgröße, KI-67 Immunzytologie und dem Auftreten perifokaler vergrößerter Lymphknoten [11, 12]. Patienten mit kleinen, 1–2 cm großen nf-PNETs haben nach operativer Entfernung signifikant bessere Überlebenschancen im Vergleich zu Patienten in einem Überwachungsprotokoll [12]. Ziele der operativen Therapie benigner Neoplasien des Pankreaskopfes sind Heilung, postoperative Symptomfreiheit, Vorbeugung der Entwicklung eines invasiven Malignoms, niedriges Risiko für operative Komplikationen und Vermeidung von Spätfolgen durch Erhaltung von Gewebe und Funktionen des Pankreas und des oberen Gastrointestinaltraktes (GI-Traktes).

## Whipple-Resektion bei benignen Pankreaskopftumoren?

Die Whipple-Resektion oder pyloruserhaltende Pankreaskopfresektion sind weltweit die chirurgischen Standardverfahren für Malignome des Pankreaskopfes und periampulläre Neoplasien. Ein hoher Grad an Standardisation der Operationstechnik, Qualität der postoperativen Intensivtherapie, Nutzung technisch hochentwickelter, interventioneller Therapieverfahren bei chirurgischen Komplikationen und Vermeidung von Spätfolgen durch institutionelle Expertise mit der komplexen Operationstechnik haben dazu geführt, die PD auch bei benignen zystischen und neuroendokrinen Tumoren als Regeleingriff anzuwenden. Whipple-Operation und PPPD (PD) sind jedoch mit erheblichen Gewebsverlusten des oberen GI-Traktes verbunden. Trotz signifikanter Senkung der Krankenhausletalität und Verminderung postoperativer Komplikationen, die Reoperation und Reintervention erfordern können, ist die PD auch bei benignen Tumoren eine komplexe Multiorganresektion mit aufklärungspflichtigem Komplikations- und Letalitätsrisiko [1]. Kliniken mit Erfahrung in der Pankreaschirurgie und großen Behandlungszahlen dokumentieren eine Krankenhausletalität von < 2 % [13, 14]. Ergebnisse nationaler und internationaler multiinstitutioneller Studien belegen, dass PD für benigne Tumoren mit einer Krankenhausletalität von 2–5 % [14–18] und einer 90-Tage-Letalität von über > 5 % assoziiert ist [19]. Spätfolgen nach PD, bedingt durch das Operationsverfahren, sind biliäre Komplikationen. Stenosen im Bereich der biliären Anastomose und rezidivierende Cholangitis werden in einer Häufigkeit von 8–13 % beobachtet [20].

Nachteile einer PD zur Therapie eines benignen Tumors sind langfristig persistierende endokrine und exokrine Dysfunktionen. Messungen der Glukosetoleranz nach PD belegen einen spätpostoperativ signifikant gestörten Glukosestoffwechsel. Daten mit hoher klinischer Evidenz zeigen, dass bei präoperativ normoglykämischen Patienten ein neu einsetzender Diabetes mellitus in 14–20 % auftritt [21, 22]; 40 % der Patienten mit präoperativ diätetisch kontrolliertem, leichtem Diabetes erfahren postoperativ eine Progression zum insulinabhängigen Diabetes. Resektion des Duodenums und der ersten Jejunumschlinge sowie Verminderung der endokrinen Kapazität durch Gewebeverlust des Pankreas, der mit der Minderung der Inselzellmasse korreliert, sind Ursachen der persistierenden endokrinen Dysfunktion [23]. Duodenektomie bewirkt eine langfristige Funktionsminderung der enteroinsularen Achse durch Verlust endokriner Zellen, die im Duodenum und oberen Jejunum residieren; die Unterbrechung der interdigestiven, gastrointestinalen Hormon- und Insulinsekretion sind Ursache der metabolischen endo- und exokrinen Dysfunktionen [23]. Nach PD tritt eine signifikante Verminderung der durch Nahrungsbolus stimulierten Freisetzung von Sekretin, Cholezystokinin, Motilin und Gastrin auf [24, 25]. Ursache der häufigen Störung der Magenentleerung nach Whipple-Resektion ist unter anderem die verminderte basale und stimulierte Motilinsekretion. Langzeitmessungen der exokrinen Funktion ergeben nach PD für benigne Tumoren bei 25–49 % der Patienten eine persistierende exokrine Stoffwechselstörung [22, 24]. Die exokrine Dysfunktion wird verursacht durch Asynchronie der Enzymsekretion im oberen Gastrointestinaltrakt und hängt vom Grad der Fibrose des Pankreasparenchyms ab. Verminderte Blutspiegel von Sekretin und Cholezystokinin, verursacht durch Duodenektomie, bewirken eine verminderte Freisetzung bikarbonatreicher Flüssigkeit und einen verminderten Transfer von Amylase, Lipase und Trypsinogen des Pankreas in den oberen Gastrointestinaltrakt. Sekretin und Cholezystokinin, beide synthetisiert in den enteroendokrinen Zellen des Duodenums und proximalen Jejunums, sind die effektiv stimulierenden Hormone für Pankreasflüssigkeit und Freisetzung von Bikarbonat [23–26]. Daten nach Langzeit-Follow-up von > 15 Jahren nach RCT mit Vergleich von PD und duodenumerhaltender Pankreaskopfresektion bei entzündlichen Tumoren des Pankreaskopfes haben gezeigt, dass als Folge einer höheren PD-assoziierten Multiorganmorbidität die klassische Resektion mit einer signifikanten Verkürzung des Überlebens um > 3 Jahre verbunden war [27]. Eine lebenslange Enzymsubstitution ist für jeden zweiten Patienten erforderlich und bewirkt eine Einschränkung der Lebensqualität nach PD. Als Folge der exokrinen und endokrinen Insuffizienz kommt es nach PD benigner Tumoren bei 20–35 % der Patienten zu einer nichtalkoholischen Steatohepatose, die das Risiko der Entwicklung einer Steatohepatitis mit nachfolgender Leberzirrhose für 5–8 % der Patienten birgt [28].

Die aktuell relevante Frage ist, ob eine PD für benigne, prämaligne zystische Neoplasien im Pankreaskopf für symptomatische oder asymptomatische Patienten als ein adäquates Therapieverfahren eingestuft werden kann [29]. Ist bei Patienten mit benignem Tumor eine PD vor dem Hintergrund der Risiken frühpostoperativer Komplikationen und spätpostoperativer, metabolischer Morbidität im Rahmen einer Entscheidungsfindung noch zu rechtfertigen [30]?

## Parenchymsparende Chirurgie bei zystischen Neoplasien

Im Lichte der Evolution lokaler Resektionsverfahren für benigne Tumoren des Pankreas haben sich Tumorenukleation (TE), duodenumerhaltende Pankreaskopfresektion (DPPHR) und Pankreasmittelsegmentresektion (PMSR) als parenchymsparende Alternativverfahren zu den klassischen Standardresektionen entwickelt. Vorteile lokaler Operationsverfahren sind Verminderung der Operationsrisiken, Minimalisierung von Gewebsverlust und Konservierung der Funktionen des oberen GI-Traktes. Die Erhaltung des Pylorus bei PD, von Traverso und Longmire 1978 in die operative Therapie eingeführt, war das erste parenchymsparende Operationsverfahren zur Verbesserung der gastrointestinalen Morbidität nach klassischer Whipple-Resektion; PPPD bewirkt Verminderung der Inzidenz des enterogastrischen Refluxes, Dumping-Syndrom und Diarrhö. TE und DPPHR haben signifikante Vorteile durch Erhaltung des Duodenums, Minimierung des Verlustes von Pankreasparenchym und Erhaltung des biliären Gangsystems.

Für benigne Tumoren des Pankreaskopfes stehen in Abhängigkeit von Größe und Dignität der Läsion zwei etablierte, jedoch bisher nicht als Standardverfahren anerkannte Operationsmethoden zur Verfügung. TE ist chirurgische Therapie der ersten Wahl für kleine Tumoren mit einem maximalen Durchmesser von 2–3 cm und wird angewandt vor allem bei nf- und f‑PNETs [31]. Bei TE kann eine Läsion des Pankreashauptganges durch die Dissektion verhindert werden durch eine intraoperative Ultraschallkontrolle der Tumorgrenze zum Pankreasgang. Nach TE werden Pankreasfisteln (POPF) mit einer Häufigkeit von 30–60 % beobachtet [32]. Das Risiko von High-volume-POPF B + C nach TE im Pankreaskopf ist deutlich erhöht, wenn einer der Pankreashauptgänge bei der Gewebsdissektion geöffnet wird. Die hohe Zahl von POPF B + C, die klinisch häufig mit einem komplizierten postoperativen Verlauf und einer verlängerten Krankenhausliegezeit und damit Kostensteigerung verbunden ist, limitiert die Anwendung von TE auf kleine Tumoren bei zystischen und neuroendokrinen Neoplasien des Pankreaskopfes. Die notwendige Dissektion der peritumoralen Lymphknoten bei neuroendokrinen Neoplasien zur Bestimmung der Dignität erfordert eine Erweiterung des operativen Vorgehens bei TE.

## Duodenumerhaltende Pankreaskopfresektion

Die DPPHR – erstmals 1969 in die Klinik eingeführt – bietet die Vorteile einer parenchymsparenden lokalen Entfernung der Neoplasie. Größe, Lokalisation und Adhärenz des Tumors zu den Nachbarorganen Duodenum und Gallenhauptgang bestimmen das Ausmaß der Gewebsresektion. Partielle oder totale DPPHR erlauben eine tumoradaptierte, individuelle Anwendung [33]. Die DPPHR ist bei entzündlichen Pankreaskopftumoren bei chronischer Pankreatitis im Vergleich zur PD als ein vorteilhaftes operatives Therapieverfahren mit hoher klinischer Evidenz durch Ergebnisse von RCTs und prospektiven, kontrollierten Studien belegt [34]. Eine Metaanalyse mit Vergleich von DPPHR und PD bei zystischen und neuroendokrinen Pankreaskopftumoren ergab für die DPPHR signifikante Vorteile durch niedrige Krankenhaussterblichkeit, geringere intra- und postoperative Bluttransfusionen, weniger Reinterventionen für frühe postoperative Komplikationen und niedrigere Reoperationsraten [13]. Die Dissektion des Pankreaskopfgewebes vom Duodenum entlang der Pankreaskapsel ist mit Erhaltung des intrapankreatischen Segmentes des Ductus choledochus (CBD) und Erhaltung der Blutversorgung des peripapillären Duodenums durch Konservierung der pankreatoduodenalen Gefäßarkaden ein zentraler operativer Schritt. Die Verminderung der arteriellen Perfusion des peripapillären Duodenums mit der Folge einer lokalen Wandischämie im peripapillären Segment des Duodenums und/oder des präpapillaren CBD stellt einen Risikofaktor der totalen DPPHR dar. Eine Ischämie der Duodenumwand ist bei 4 von 729 Patienten nach DPPHR beobachtet worden und machte eine Konversion zur totalen DPPHR mit Resektion des peripapillären Duodenums mit End-zu-End-Anastomosierung notwendig [33, 35]. Ein Vergleich der chirurgischen Technik zeigt, dass die totale DPPHR chirurgisch-technisch bis auf die Dissektion des Pankreaskopfes zwischen Serosa des Duodenums und der Kapsel des Pankreas vergleichbare operative Schritte erfordert wie bei klassischer PD. Das Risiko der Entwicklung von Pankreasfisteln ist nach totaler DPPHR und PD gleich hoch. Komplikationen am biliären System, die zu interventionellen Eingriffen führen, sind nach *partieller* DPPHR signifikant weniger häufig als nach PD [13]. Zur Vermeidung von Verletzungen des biliären Systems bei totaler DPPHR ist eine intraoperative Darstellung des Gallengangs mittels Indocyaningrünfluoreszenz sinnvoll und bewirkt eine Verminderung von Verletzungen des intrapankreatischen CBD. Das Sampling lokaler Lymphknoten zur Feststellung der Dignität eines neuroendokrinen Tumors ist bei DPPHR ohne wesentliche Erweiterung der Operation möglich. Ergebnisse von Studien nach laparoskopischer und roboterassistierter totaler DPPHR belegen, dass bei minimal-invasiven Operationsverfahren zusätzliche Vorteile in Bezug auf Blutverlust und Krankenhausliegezeiten erreicht werden können [35]. Wie Langzeitbeobachtungen zeigen, ist das Risiko eines lokalen Rezidivs an der Anastomose nach DPPHR wie nach PD mit < 3 % gleich niedrig [36]. Ergebnisse prospektiver, kontrollierter Studien haben gezeigt, dass die endo- und exokrinen Pankreasfunktionen nach DPPHR erhalten bleiben [21]. Patienten mit präoperativ normalen endo- und exokrinen Pankreasfunktionen haben nach DPPHR selten einen neu auftretenden Diabetes mellitus und eine normale exokrine Funktion. Die Vermeidung der metabolischen Morbidität hat dazu geführt, dass bei Jugendlichen und Kindern, die prädominant an einer SPN erkranken, die DEPKR *als vorteilhafte operative Therapie in der Kinderchirurgie* Anerkennung gefunden hat [37].

## Fazit

Die Whipple-Resektion oder PPPD benigner zystischer Neoplasien des Pankreaskopfes sind assoziiert mit dem erheblichen Risiko chirurgischer Komplikationen und einer Krankenhausletalität bis zu 5 %. Tumorenukleation und duodenumerhaltende Pankreaskopfresektion sind parenchymsparende lokale Resektionsverfahren, die mit einer signifikant niedrigen Operationsmorbidität sowie Erhaltung der endo- und exokrinen Pankreasfunktionen assoziiert sind. Tumorenukleation und duodenumerhaltende Pankreaskopfresektion sind vorteilhafte operative Alternativverfahren zur PD.
